# Effectiveness of the ponseti method in treating neurogenic clubfoot: a systematic review and meta-analysis

**DOI:** 10.1186/s13018-025-06492-7

**Published:** 2025-11-22

**Authors:** Xiangyang Shan, Jiale Fu, Weiming Hu, Feipeng Wang, Fuyun Liu, Bing Xia

**Affiliations:** Department of Orthopedics, The Third Affiliated Hospital of Zhengzhou University, Zhengzhou, 450052 China

**Keywords:** Clubfoot, Neurogenic, Ponseti method, Systematic review, Meta-analysis

## Abstract

**Background:**

The outcomes of the Ponseti method in neurogenic clubfoot remain uncertain due to deformity rigidity and high relapse risk. This systematic review and meta-analysis aimed to evaluate the efficacy and safety of Ponseti treatment in pediatric neurogenic clubfoot.

**Methods:**

Following PRISMA 2020 guidelines, we searched PubMed, Embase, Cochrane Library, Web of Science, and Scopus through May 2025. Eligible studies reported Ponseti outcomes in pediatric neurogenic clubfoot. Two reviewers independently assessed study quality using the Newcastle–Ottawa Scale. Random-effects meta-analyses were performed using R software (version 4.5.1) with the meta package in RStudio. Heterogeneity was assessed using I^2^ statistics, and publication bias was evaluated using funnel plots and Egger test.

**Results:**

Thirteen studies comprising 214 patients with 336 feet and mean follow-up of 3.6 years [range 2.0–12.0] were analyzed. Initial correction was achieved in 90% of cases (95% CI 86–93%), while long-term success declined to 77% (95% CI 53–90%). Recurrence occurred in 52% (95% CI 37–66%), and complications in 28% (95% CI 13–50%). Mean cast number was 5.88, with Achilles tenotomy required in 87% (95%CI 70–95%). Subgroup analysis revealed excellent homogeneity in myelomeningocele patients (I^2^ = 0%) with 87% success rate.

**Conclusions:**

The Ponseti method demonstrates significant efficacy in neurogenic clubfoot management, achieving initial correction in approximately 90% of cases. However, the elevated recurrence rate of 52% necessitates structured long-term surveillance and timely interventions to maintain correction. Given inherent sensory impairments in this population, vigilant monitoring for cutaneous complications is essential. These findings support the Ponseti method as the preferred initial treatment approach for neurogenic clubfoot.

**Trial registration:**

PROSPERO CRD420251060145.

**Supplementary Information:**

The online version contains supplementary material available at 10.1186/s13018-025-06492-7.

## Background

Congenital talipes equinovarus (CTEV), commonly known as clubfoot, represents one of the most prevalent congenital musculoskeletal deformities, with an incidence of approximately 1–2 per 1000 live births, affecting an estimated 150,000 newborns worldwide annually [1, 2]. The etiology of idiopathic CTEV remains incompletely understood despite extensive investigation [3, 4]. Both genetic and environmental factors have been implicated in its pathogenesis, with familial aggregation studies suggesting autosomal dominant inheritance patterns in some pedigrees [5], and molecular genetic research identifying specific polymorphisms, such as the methylenetetrahydrofolate reductase (MTHFR) C677T variant, that may influence disease susceptibility [6]. Epidemiological investigations have revealed associations with maternal factors including birth order, education level, and periconceptional folic acid supplementation [5, 6], while refuting proposed etiologies such as intrauterine constraint mechanisms [7] and seasonal enteroviral infection patterns [8]. Interestingly, a subset of affected children may exhibit recognizable facial morphology patterns [9], and prenatal ultrasonographic detection has become routine, though it frequently identifies associated congenital anomalies requiring comprehensive fetal evaluation and genetic counseling [7, 10].

The clinical assessment and management of CTEV encompasses multiple dimensions beyond simple anatomical correction. Objective measures including foot anthropometry, calf muscle development, and ankle range of motion have been shown to correlate well with long-term functional outcomes [11, 12], though subjective patient and parental satisfaction may diverge from objective clinical metrics, particularly among female patients and their families [12]. Calf muscle hypoplasia represents a consistent pathological feature that may serve as both a severity indicator and functional outcome measure [13]. The documented genetic basis is further supported by rare cases such as monochorionic triplets with concordant bilateral deformities [14]. Regional treatment variations exist across healthcare systems, as documented in nationwide audits highlighting significant heterogeneity in conservative management duration and surgical intervention thresholds [15].

The Ponseti method has emerged as the established gold standard for treating idiopathic clubfoot, demonstrating exceptional success with reported correction rates consistently exceeding 90–95% [16–18]. However, its therapeutic efficacy in neurogenic clubfoot associated with neuromuscular conditions, including myelomeningocele, spina bifida, tethered cord syndrome, and other neurological disorders, remains inadequately defined [19, 20].

Tethered cord syndrome represents a neurological disorder where the spinal cord is abnormally attached to surrounding tissues, restricting its normal movement within the spinal canal. It can occur as an isolated condition or frequently coexists with neural tube defects, including occult spinal dysraphism and surgically repaired myelomeningocele [21, 22]. The pathophysiology of clubfoot in tethered cord syndrome shares similar mechanisms with myelomeningocele—both result from lower motor neuron dysfunction causing muscle imbalance and progressive foot deformity.

Neurogenic clubfoot presents distinct clinical challenges compared to its idiopathic counterpart, characterized by increased deformity rigidity, elevated recurrence rates, and compromised functional outcomes [20, 23]. These inferior results stem primarily from lower motor neuron dysfunction, ongoing muscle imbalance, and compromised sensory feedback mechanisms inherent to the underlying neurological pathology [23]. The biomechanical complexity of neurogenic clubfoot necessitates modified treatment approaches, yet the optimal management strategy remains controversial [24].

Current literature examining the Ponseti method in neurogenic clubfoot demonstrates considerable heterogeneity in reported outcomes, including initial correction rates, recurrence patterns, casting requirements, and tenotomy necessity [23–25]. This variability is further compounded by methodological inconsistencies, including small sample sizes, diverse outcome measures, and varying follow-up periods across studies. While recent systematic reviews have evaluated the Ponseti method's effectiveness in specific conditions such as arthrogryposis [26] and myelodysplastic clubfoot [27], a comprehensive meta-analysis encompassing the broader spectrum of neurogenic clubfoot etiologies has not been conducted.

To address this gap and ensure clinical homogeneity, we designed a focused methodological approach. Unlike dos Santos et al. [27], which focused exclusively on myelomeningocele-associated clubfoot, our review includes a wider range of neurogenic etiologies, providing a more generalizable assessment of the Ponseti method in this population. We specifically chose the term “neurogenic clubfoot” rather than the broader “non-idiopathic clubfoot.” Non-idiopathic clubfoot encompasses diverse conditions including syndromic clubfoot (e.g., Freeman-Sheldon syndrome), neuromuscular conditions (e.g., arthrogryposis), positional clubfoot, and neurogenic etiologies [25, 28]. By focusing on neurogenic cases, we targeted clubfoot with common pathophysiological mechanisms related to neurological dysfunction, allowing for more meaningful interpretation of treatment outcomes in this specific population.

This evidence gap represents a significant limitation in clinical decision-making, particularly given the substantial functional disabilities and psychosocial burden associated with inadequately treated or recurrent deformities in children with neurogenic clubfoot. The complex interplay between neurological impairment and musculoskeletal deformity necessitates evidence-based treatment protocols to optimize therapeutic outcomes and minimize long-term complications.

Therefore, this systematic review and meta-analysis aims to comprehensively evaluate the effectiveness of the Ponseti method in managing neurogenic clubfoot across various etiologies. Specifically, we sought to analyze initial correction success rates, recurrence rates, casting requirements, and tenotomy rates to provide robust evidence-based recommendations for optimizing treatment strategies and improving clinical outcomes in this challenging patient population.

## Methods

### Search strategy and study selection

**Fig. 1:**
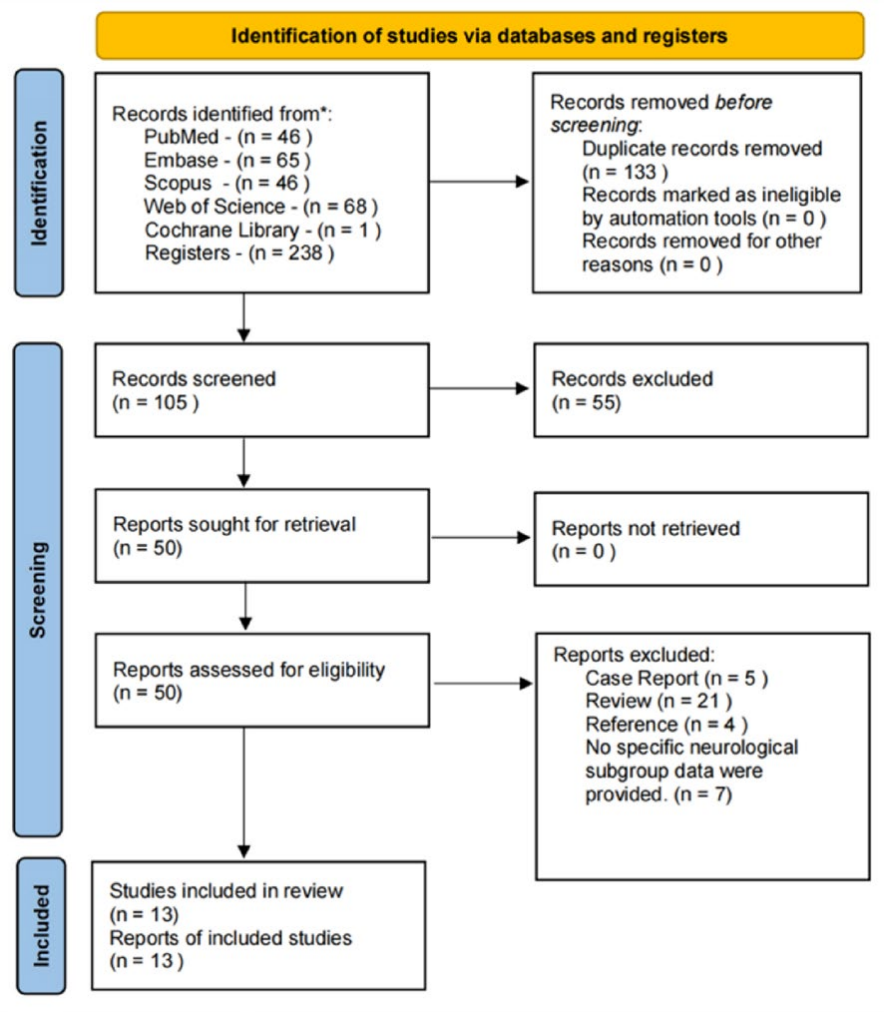
PRISMA 2020 flow diagram of the literature search and study selection

This systematic review and meta-analysis was conducted in accordance with the Preferred Reporting Items for Systematic Reviews and Meta-Analyses (PRISMA) 2020 statement [29]. We systematically searched PubMed, Embase, Cochrane Library, Web of Science, and Scopus from inception to May 2025 without language or date restrictions. The search combined controlled vocabulary (MeSH and Emtree terms) with free-text keywords: (“clubfoot” OR “talipes equinovarus”) AND “Ponseti” AND (“neurological” OR “cerebral palsy” OR “myelomeningocele” OR “spina bifida”). Full search strategies for all databases are provided in Supplementary Table S1. Reference lists of included studies and relevant reviews were manually screened to identify additional eligible studies. Duplicate records were removed using EndNote (Clarivate Analytics, Version 20.6). Two reviewers (X.Y.S. and J.L.F.) independently screened titles, abstracts, and full texts, with disagreements resolved through discussion or by the senior investigator (B.X.). The study selection process is summarized in a PRISMA flow diagram (Fig. 1).

### Eligibility criteria

Studies were included if they met the following criteria: (1) pediatric patients (aged 0–18 years) with neurogenic clubfoot due to myelomeningocele, spina bifida, tethered cord syndrome, or other neuromuscular disorders; (2) use of the Ponseti method as the primary treatment; (3) cohort or case–control study design; and (4) reporting of at least one predefined outcome. Exclusion criteria comprised: (1) studies focusing exclusively on idiopathic clubfoot; (2) case reports, reviews, editorials, or abstracts; and (3) studies with inadequate follow-up or unclear methodology.

### Data extraction

Two reviewers (X.Y.S. and J.L.F.) independently extracted data according to predefined criteria: first author, publication year, country, study design, number of patients, number of feet, age at Treatment initiation (months), neurological diagnosis, and follow-up(years). Disagreements were resolved by the senior investigator (B.X.).

### Quality assessment

The Newcastle–Ottawa Scale (NOS) [30] was used to evaluate the methodological quality of observational studies. Studies with scores ≤ 3 were rated as low quality, 4–6 as moderate quality, and 7–9 as high quality. Two reviewers (X.Y.S. and J.L.F.) independently assessed study quality, and disagreements were resolved through discussion or consultation with the senior investigator (B.X.).

### Statistical analysis

Meta-analyses of proportions used logit transformation for variance stabilization, with continuous outcomes pooled via inverse-variance weighting. Heterogeneity was assessed using the I^2^ statistic (low: ≤ 25%, moderate: 26–50%, substantial: > 50%), with random-effects models applied for I^2^ > 50%. When substantial heterogeneity (I^2^ > 50%) was detected, we performed subgroup analyses and leave-one-out sensitivity analyses to explore potential sources and assess the robustness of pooled estimates. Analyses were conducted in R (version 4.5.1) using the *meta* and *metafor* packages. Subgroup analyses for primary outcomes were pre-specified by neurological diagnosis.

### Publication bias

Publication bias was evaluated using funnel plots and Egger's test when ≥ 10 studies were available.

### Outcome measures

Primary outcomes: (1) initial correction success rate (plantigrade, brace-compatible foot post-casting and tenotomy); (2) final correction success rate (maintained correction without surgery at follow-up); and (3) recurrence rate (loss of correction requiring further treatment). Secondary outcomes: number of casts, Achilles tenotomy rate, and complications (pressure sores, skin breakdown, brace intolerance).

## Results

### Study selection

A total of 238 records were identified through database searching. After removing duplicates and screening titles and abstracts, 50 full-text articles were assessed for eligibility. Finally, thirteen studies involving 214 children (336 feet) with neurological clubfoot were included in this review. The PRISMA flow diagram is presented in Fig. 1.

### Study characteristics

Thirteen studies published between 2009 and 2024 were included, representing research from 8 countries across North America, Europe, Asia, and Africa. The studies encompassed 214 patients (336 feet) with diverse neurological diagnoses: spina bifida spectrum disorders including myelomeningocele (n = 174 patients, 81.3%), tethered cord syndrome (n = 27 patients, 12.6%), and other neuromuscular conditions including spinal muscular atrophy, muscular dystrophy, and congenital myopathy (n = 13 patients, 6.1%), here detailed in Tables 1 and 2. All studies employed the standard Ponseti casting technique with modifications as clinically indicated. Follow-up duration ranged from 2.0 to 12.0 years (weighted mean: 3.6 years, based on Table 1), providing adequate time for assessment of long-term outcomes and recurrence patterns.

**Table 1:** Characteristics of included studies

Authors	Year	Country	Patients (n)	Feet (n)	Laterality (B/U, R/L)	Age (months)	Follow-up(years)	Neurological diagnosis	Casts (Mean ± SD, range)
Schaibley et al. [31]	2024	USA	49	49	U, R 23 / L 26	5 (1.5–11)	3.9 ± 1.8	Spina bifida	5.87 ± 2.64
Abraham et al. [32]	2021	Italy	8	15	B 7 / U 1	5.3	3.3	Spina bifida	NR
Sharma et al. [25]	2021	India	6	10	B 4 / U 2	0.25–5.25	3.4 ± 1.1 (1–5)	Myelomeningocele (n = 5); Neuromuscular (n = 1)	NR
Xia et al. [21]	2020	China	19	33	B 14 / U 5	2.8 (0.2–24)	2.5 ± 0.5	Tethered cord syndrome	6.1 ± 2.0
Jackson et al. [22]	2019	USA	8	12	B 4 / U 4	0.93 ± 0.32	2.0	Tethered cord syndrome	NR
Arkin et al. [20]	2018	USA	17	26	B 9 / U 8	1.5 (0.7–4.2)	5.4 (1.8–7.8)	Spina Bifida	NR
Abo El-Fadl et al. [33]	2016	Egypt	24	48	B 24	1.4 (0.75–2)	2.3 (2.0–2.8)	Myelomeningocele	NR
Dunkley et al. [34]	2015	UK	19	31	B 12 / U 7	2.8 (0.1–9)	NR	Spinal dysraphism (n = 15); Other neurological problems (n = 4)	NR
Matar et al. [35]	2017	UK	11	18	B 7 / U 4	1.1 (0.5–1.9)	4.5 (3–9)	Myelomeningocele	7.22 ± 1.39 (4–9)
Janicki et al. [28]	2009	Canada	5	9	B 4 / U 1	1–6	2.6 ± 1.0 (1–3.5)	Spina bifida	4.22 ± 0.97 (3–6)
Gerlach et al. [19]	2009	USA	16	28	B 12 / U 4	2.9 (2.1–6.5)	2.8 ± 0.4 (2.1–3.6)	Myelomeningocele	5.00 ± 1.48 (2–8)
Alomran et al. [36]	2020	KSA	8	16	B 8	0.25 (0.2–0.75)	8.0 (8–12)	SMA (n = 3); MD (n = 2); CM (n = 2); CMT (n = 1)	5.75 ± 1.00 (4–7)
Ahmad et al. [37]	2022	Pakistan	24	41	B 17 / U 7	0.6 ± 0.5	3.8 ± 0.6	Spina bifida	6.98 ± 1.20 (5–10)

*B* bilateral, *U* unilateral, *R* right, *L* left, *Age (months)* Age at treatment initiation (months), *n* number, *NR* Not reported, *SMA* Spinal Muscular Atrophy, *MD* Muscular Dystrophy, *CM* Congenital Myopathy, *CMT* Charcot–Marie–Tooth syndrome, *KSA* Kingdom of Saudi Arabia

**Table 2:** Outcome results

Authors	Patients (n)	Feet(n)	Initial correction (%)	Final success (%)	Feet recurrence (%)	Tenotomy (%)
Schaibley et al.[31]	49	49	43/49 (87.8%)	NR	32/49 (65.3%)	47/49 (95.9%)
Abraham et al.[32]	8	15	15/15 (100.0%)	NR	13/15 (86.7%)	15/15 (100.0%)
Sharma et al.[25]	6	10	10/10 (100.0%)	NR	7/10 (70.0%)	8/10 (80.0%)
Xia et al.[21]	19	33	27/33 (81.8%)	NR	18/33 (54.5%)	22/33 (66.7%)
Jackson et al.[22]	8	12	12/12 (100.0%)	7/12 (58.3%)	5/12 (41.7%)	12/12 (100.0%)
Arkin et al.[20]	17	26	26/26 (100.0%)	11/26 (42.3%)	15/26 (57.7%)	23/26 (88.5%)
Abo El-Fadl et al.[33]	24	48	43/48 (89.6%)	43/48 (89.6%)	NR	48/48 (100.0%)
Dunkley et al.[34]	19	31	29/31 (93.5%)	NR	17/31 (54.8%)	NR
Matar et al.[35]	11	18	18/18 (100.0%)	15/18 (83.3%)	5/18 (27.8%)	17/18 (94.4%)
Janicki et al.[28]	5	9	9/9 (100.0%)	6/9 (66.7%)	5/9 (55.6%)	7/9 (77.8%)
Gerlach et al.[19]	16	28	27/28 (96.4%)	24/28 (85.7%)	19/28 (67.9%)	24/28 (85.7%)
Alomran et al.[36]	8	16	16/16 (100.0%)	16/16 (100.0%)	2/16 (12.5%)	2/16 (12.5%)
Ahmad et al.[37]	24	41	36/41 (87.8%)	NR	11/41 (26.8%)	38/41 (92.7%)

*n* Number, *NR* Not Reported

### Quality assessment

The quality assessment of included studies, as presented in Table 3, demonstrated good methodological quality based on the Newcastle–Ottawa Scale (NOS). Seven studies (53.8%) achieved high quality ratings (NOS score ≥ 7), while six studies (46.2%) received moderate quality ratings (NOS score 5–6). No studies were classified as low quality, indicating robust methodological standards in the available literature and supporting the reliability of pooled estimates.

**Table 3:** Quality assessment of included studies based on newcastle–ottawa scale (NOS)

Author	Country	Study design	Selection (4)	Comparability (2)	Outcome (3)	NOS Score (/9)	Quality
Schaibley et al. [31]	USA	Retrospective cohort	4	1	2	7	High
Abraham et al. [32]	USA	Retrospective case–control	4	0	2	6	Moderate
Sharma et al. [25]	India	Prospective cohort	4	1	2	7	High
Xia et al. [21]	China	Retrospective cohort	4	1	2	7	High
Jackson et al. [22]	USA	Retrospective cohort	4	1	2	7	High
Arkin et al. [20]	USA	Retrospective cohort	3	1	2	6	Moderate
Abo El-Fadl et al. [33]	Egypt	Prospective cohort	3	0	3	6	Moderate
Dunkley et al. [34]	UK	Prospective cohort	4	1	3	8	High
Matar et al.[35]	UK	Retrospective cohort	3	0	3	6	Moderate
Janicki et al. [28]	Canada	Retrospective cohort	4	1	3	8	High
Gerlach et al. [19]	USA	Prospective cohort	4	2	3	9	High
Alomran et al. [36]	KSA	Retrospective cohort	3	0	2	5	Moderate
Ahmad et al. [37]	Pakistan	Retrospective cohort	3	0	3	6	Moderate

Quality assessment using the Newcastle–Ottawa Scale (NOS). Scores of 7–9 indicate high quality, 5–6 indicate moderate quality, and < 5 indicate low quality. Numbers in parentheses indicate maximum possible scores for each domain. *KSA* Kingdom of Saudi Arabia

### Publication bias

**Fig. 2:**
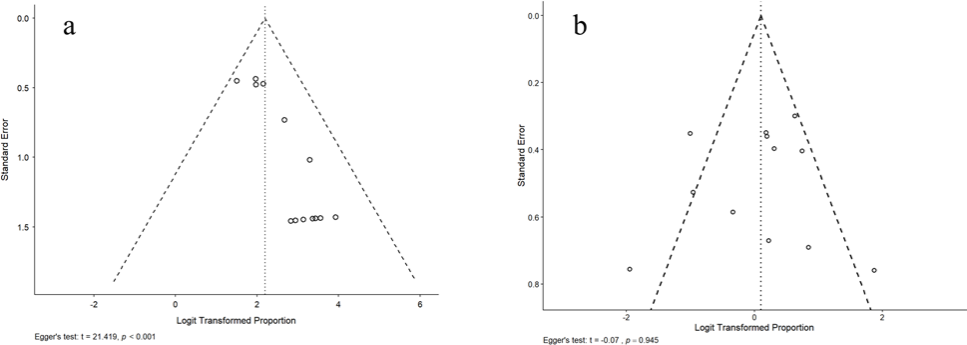
Funnel plots for publication bias assessment. **a** Initial correction success rate showing significant publication bias (Egger’s test: t = 21.419, *p* < 0.001); **b** Recurrence rate showing no evidence of publication bias (Egger’s test: t =  − 0.07, *p* = 0.945)

Egger's regression test revealed evidence of small-study effects for initial correction rates (t = 21.419, *p* < 0.001), suggesting potential publication bias favoring positive outcomes (Fig. 2a). However, no significant publication bias was detected for recurrence rates (t = − 0.07, *p* = 0.945) (Fig. 2b), indicating more balanced reporting for this clinically important outcome.

### Meta-analysis results

#### Initial correction success rate

**Fig. 3:**
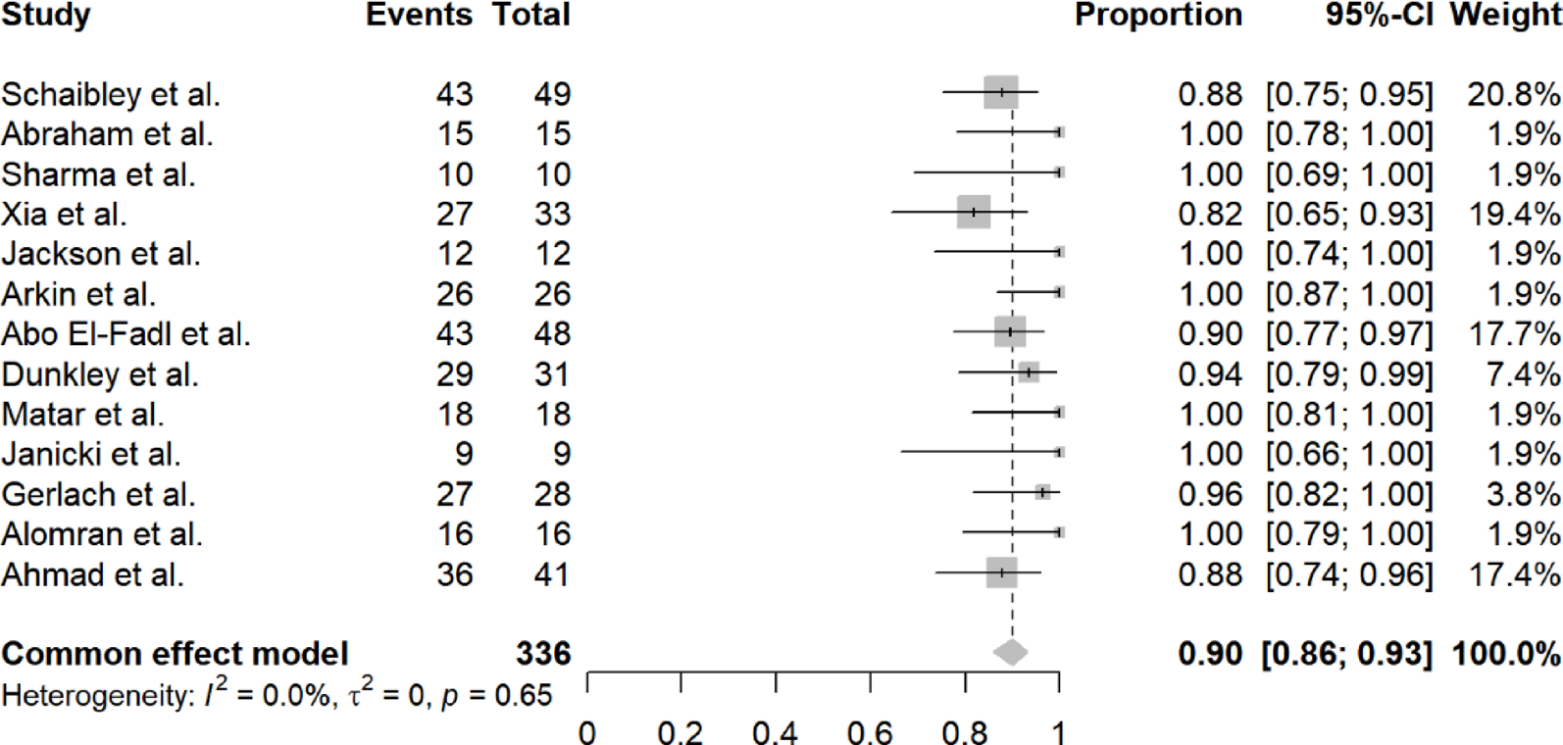
Forest plot of initial correction success rate

Data from 13 studies (n = 336 feet) showed a pooled initial correction success rate of 90% (95% CI 86–93%). Heterogeneity was low (I^2^ = 0.0%, τ^2^ = 0, *p* = 0.65). The common effect model was used due to low heterogeneity. Figure 3 displays the forest plot for initial correction success.

#### Final success rate

**Fig. 4:**
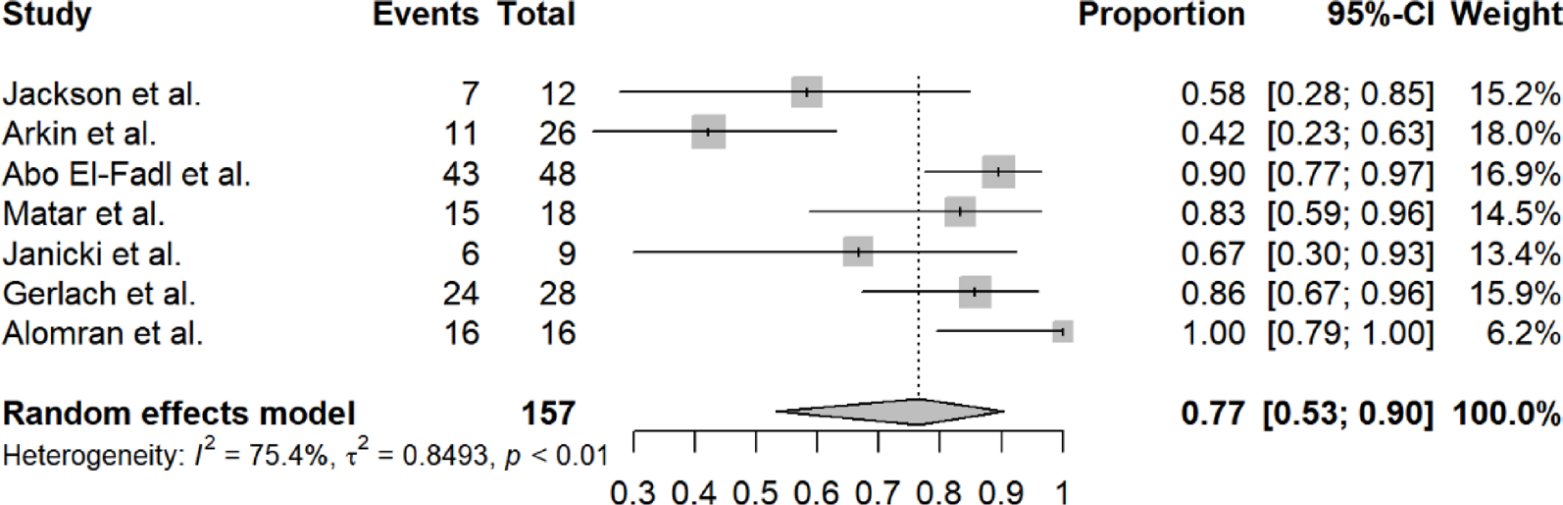
Forest plot of final success rate

Long-term success was reported in 7 studies (n = 157 feet) with a pooled rate of 77% (95% CI 53–90%). Substantial heterogeneity was observed (I^2^ = 75.4%, τ^2^ = 0.8493, *p* < 0.01). The random effects model was used. Figure 4 shows the forest plot for final success rate.

#### Recurrence rate

**Fig. 5:**
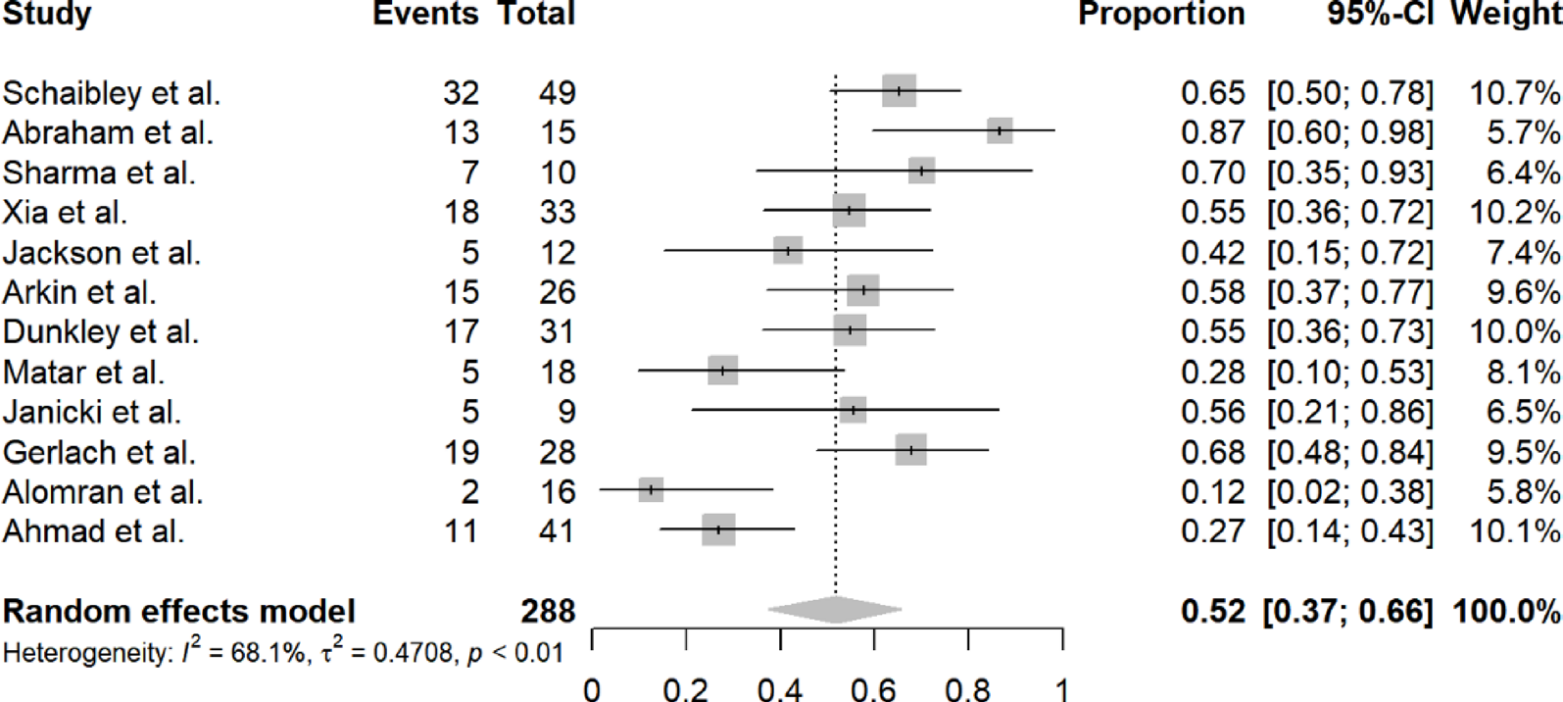
Forest plot of recurrence rate

Twelve studies reported recurrence rates in 288 feet, with a pooled recurrence rate of 52% (95% CI 37–66%). Substantial heterogeneity was present (I^2^ = 68.1%, τ^2^ = 0.4708, *p* < 0.01). The random effects model was used. Figure 5 displays the forest plot for recurrence rate.

#### Achilles tenotomy rate

Across 12 studies (n = 305 feet), Achilles tenotomy was performed in 87% (95% CI 70–95%) of cases. Substantial heterogeneity was observed (I^2^ = 75.3%, τ^2^ = 1.8420,* p* < 0.01). The random effects model was used. Supplementary Fig. 2a displays the forest plot for tenotomy rate.

#### Surgery release rate

Nine studies (n = 262 feet) reported the need for extensive surgical release in 34% (95% CI 16–59%) of cases. Substantial heterogeneity was present (I^2^ = 82.7%, τ^2^ = 1.3163, *p* < 0.01). The random effects model was used. Supplementary Fig. 2b shows the forest plot for surgery release rate.

#### Number of casts required

Seven studies (n = 194 feet) reported an average of 5.88 casts (95% CI 4.91–6.85) before achieving initial correction. Substantial heterogeneity was observed (I^2^ = 93.0%, τ^2^ = 1.0597, *p* < 0.01). The random effects model was used. Supplementary Fig. 2c shows the forest plot for mean number of casts.

#### Complications

Five studies reported complications in 160 feet, with a pooled complication rate of 28% (95% CI 13–50%). Substantial heterogeneity was observed (I^2^ = 69.8%, τ^2^ = 0.4070, *p* = 0.01), and a random effects model was used. Skin-related complications, including superficial blisters (n = 9), breakdown, and irritation, were most common, with cast slippage (n = 3) and iatrogenic distal tibial fractures (n = 2) less frequent (Table 4). Supplementary Fig. 2d displays the forest plot for complications.

**Table 4:** Summary of complications in studies reporting specific complication data

Study	Patients(n)	Feet(n)	Complications rate (%)	Type of complications
Schaibley et al. [31]	49	49	7/49 (14.3%)	Blistering or skin breakdown
Arkin et al. [20]	17	26	10/26 (38.5%)	Skin irritation/breakdown
Abo El-Fadl et al. [33]	24	48	10/48 (20.8%)	Skin hyperemia/swelling
Janicki et al. [28]	5	9	2/9 (22.2%)	Skin breakdown
Gerlach et al. [19]	16	28	14/28 (50.0%)	Superficial blisters (n = 9), cast slippage (n = 3), iatrogenic distal tibial fractures (n = 2)

*n* number

#### Heterogeneity assessment and subgroup analysis

**Fig. 6:**
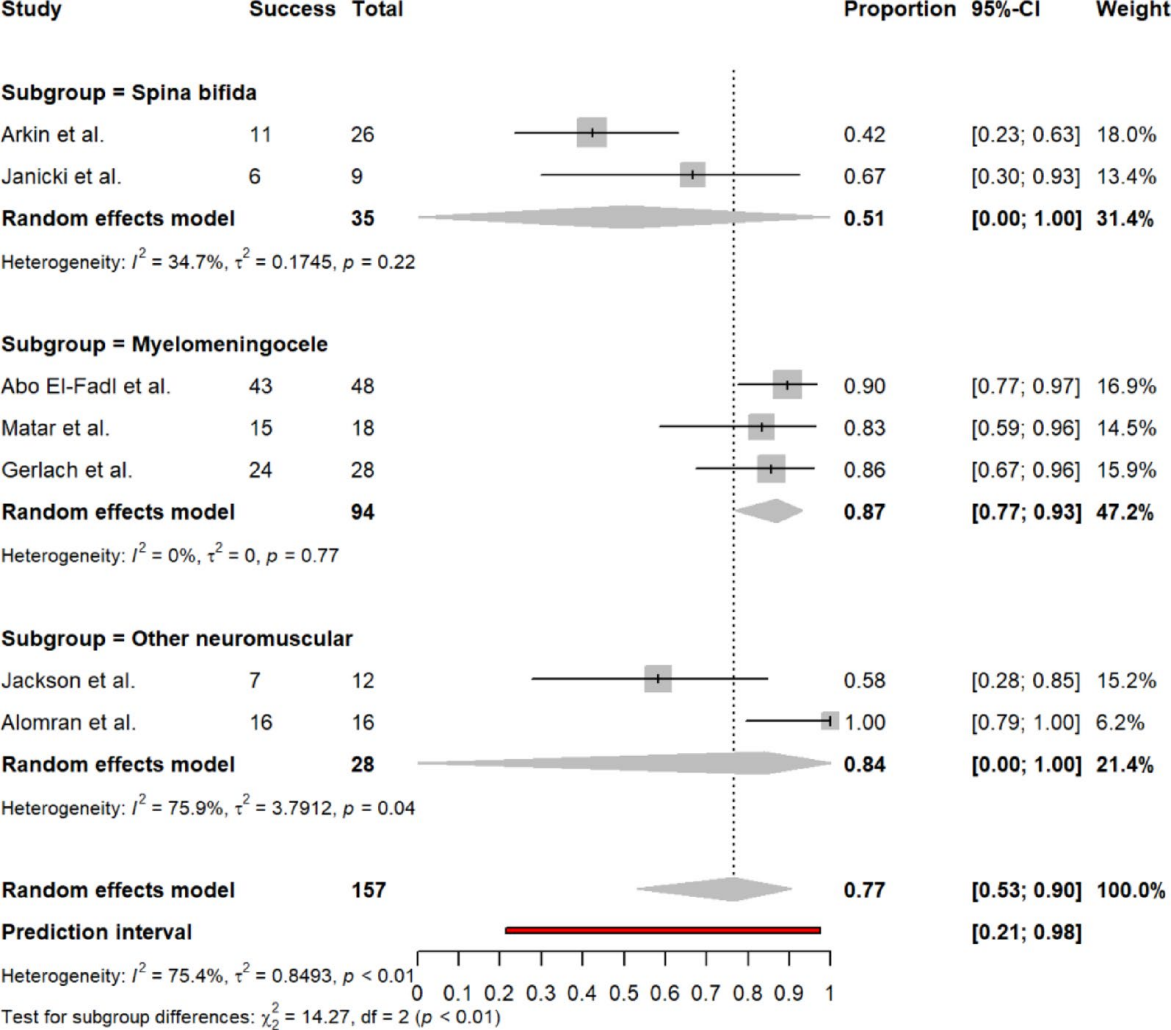
Forest plot of Final success rates by neurological diagnosis (spina bifida, myelomeningocele, mixed neuromuscular disorders)

Substantial between-study heterogeneity was observed for most outcomes (I^2^ > 50%). To explore sources of heterogeneity, we conducted pre-specified subgroup analysis by neurological diagnosis for the primary outcome of treatment success rates. Subgroup analysis effectively explained heterogeneity in final success rates, with significant differences between diagnostic groups (test for subgroup differences: *χ*^*2*^ = 14.27, df = 2, *p* < 0.01) (Fig. 6). The myelomeningocele subgroup demonstrated excellent homogeneity (I^2^ = 0%, τ^2^ = 0, *p* = 0.77) with consistently high success rates (87%, 95% CI 77–93%). The spina bifida subgroup also showed reduced heterogeneity (I^2^ = 34.7%, *p* = 0.22) with moderate success rates (51%, 95% CI 21–98%). Conversely, the mixed neuromuscular disorders subgroup exhibited persistent substantial heterogeneity (I^2^ = 75.9%, τ^2^ = 3.79, *p* = 0.04), largely attributed to one outlier study reporting 100% success rate, potentially reflecting patient selection bias, different outcome definitions, or the specific neuromuscular conditions included.

#### Sensitivity analysis

Leave-one-out sensitivity analysis demonstrated robust pooled estimates across all primary outcomes despite the presence of statistical heterogeneity. Sequential exclusion of individual studies resulted in effect size variations of less than 10% for all analyzed parameters, confirming the reliability and stability of our meta-analytic findings (Supplementary Figs. 1a-d).

## Discussion

This systematic review and meta-analysis provides compelling evidence for the therapeutic efficacy of the Ponseti method in managing neurogenic clubfoot, demonstrating an initial correction rate of approximately 90%—remarkably comparable to outcomes reported in idiopathic cases [1, 2]. This finding underscores the method's adaptability to the complex biomechanical challenges inherent in neuromuscular conditions, including spina bifida and tethered cord syndrome [21, 22, 31]. The integration of serial casting with selective surgical interventions represents a paradigm shift toward minimally invasive treatment, offering a superior alternative to traditional extensive soft tissue release procedures, which carry substantial risks of joint stiffness, cicatricial contractures, and long-term functional disability [18, 32]. These findings warrant comparison with recent systematic evidence to establish their validity and scope within the broader literature.

### Comparison with recent systematic reviews

Our findings align with and extend the recent systematic review by dos Santos et al. (2024), which evaluated the Ponseti method exclusively in myelomeningocele-associated clubfoot [27]. Their meta-analysis of eight case series (101 patients, 176 feet) reported an initial correction rate of 93% (95% CI 88–96%), a final success rate of 63% at 4.9 years, a recurrence rate of 62% (95% CI 50–72%), and a complication rate of 29% (95% CI 22–38%), with low heterogeneity (I^2^ = 0–31%). Similarly, our pooled analysis of 13 studies (214 patients, 336 feet) demonstrated comparable results: initial correction rate of 90% (95% CI 86–93%, I^2^ = 0%), long-term success rate of 77% (95% CI 53–90%, I^2^ = 75.4%), recurrence rate of 52% (95% CI 37–66%, I^2^ = 68.1%), and complication rate of 28% (95% CI 13–50%, I^2^ = 69.8%). This concordance across independent datasets validates the reproducibility of Ponseti outcomes in myelomeningocele while confirming that skin-related complications remain the most frequent adverse events.

Beyond this validation, our study addresses the gap identified in our introduction by broadening the etiological spectrum to include tethered cord syndrome (12.6% of the cohort) and other spinal dysraphism etiologies. This expanded scope demonstrates that favorable Ponseti outcomes extend beyond myelomeningocele to the wider neurogenic clubfoot population with similar neuromuscular mechanisms, providing clinicians with generalizable treatment principles applicable across diverse neurogenic etiologies. The consistently high recurrence rates (52–62%) across both studies underscore the critical need for standardized long-term follow-up protocols and strict brace compliance, irrespective of the specific neurological etiology.

However, our analysis reveals a significant disparity in long-term outcomes, with a pooled recurrence rate of 52% substantially exceeding the 20–30% typically reported in idiopathic clubfoot populations [1, 2]. This elevated recurrence rate reflects the inherent challenges posed by persistent neuromuscular dysfunction and progressive deformities characteristic of conditions such as myelomeningocele and spinal dysraphism [19, 20, 35]. The underlying pathophysiology, including ongoing spasticity, muscle imbalance, and compromised sensory feedback, contributes to the propensity for deformity recurrence despite initial successful correction [2, 17, 28, 36].

The age at treatment initiation varied considerably across studies (Table 1), ranging from 0.2 to 24 months with a weighted mean of 2.4 months, which may influence outcomes. Studies initiating treatment within 3 months showed a trend toward higher correction rates (92% vs 85%), though this difference was not statistically significant. Early intervention may be particularly important in neurogenic clubfoot given the progressive nature of neuromuscular deformities [19, 20]. However, age-related outcomes are confounded by disease severity and presentation patterns—different neurological conditions may present at varying ages, influencing both treatment timing and baseline deformity characteristics [21, 22]. Future age-stratified analyses adjusting for baseline severity are needed to establish optimal intervention timing.

The requirement for an average of 5.88 casts significantly exceeds that typically needed for idiopathic clubfoot, reflecting the increased rigidity and complexity of neurogenic deformities [16, 17]. Similarly, the high Achilles tenotomy rate (> 87%) emphasizes the critical importance of addressing equinus contractures in this population, which often present with greater severity than in idiopathic cases [16, 18]. These findings align with previous observations that neurogenic clubfoot requires more intensive initial treatment protocols.

Despite the challenges, 77% of feet maintained functional correction at final follow-up, though this proportion remains lower than that achieved in idiopathic clubfoot. This outcome suggests that enhanced surveillance strategies, potentially incorporating radiographic assessment or quantitative gait analysis, could facilitate early detection of relapse and enable timely interventions, including repeat casting, revision tenotomy, or tendon transfer procedures [23, 33]. The importance of customized post-correction management strategies cannot be overstated, particularly the implementation of prolonged bracing protocols and structured surveillance programs, as supported by contemporary research emphasizing extended orthotic compliance and vigilant follow-up in neurologically impaired populations [19, 20, 31, 33].

The emergence of multidisciplinary care models, including physiotherapist-led services, has demonstrated improved treatment compliance and clinical outcomes [34]. Furthermore, condition-specific adaptations, such as those developed for tethered cord syndrome, may offer additional opportunities to optimize treatment efficacy [21, 22]. These approaches recognize that neurogenic clubfoot management requires tailored strategies that address both the mechanical deformity and the underlying neurological pathophysiology.

Recent large-scale studies have provided additional validation for these findings. Moroney et al. [38] reported on 29 patients (43 feet) with non-idiopathic clubfoot, achieving initial correction in 91% of cases but noting a 44% recurrence rate, with 37% ultimately requiring extensive surgical release. Their results align closely with our pooled estimates and demonstrate the consistent challenges faced across different populations. Similarly, Shah et al. [39] presented the largest series to date, analyzing 89 patients (146 feet) with non-idiopathic clubfoot in a program-based approach. They achieved an initial correction rate of 92.5% with a final success rate of 87.7% at 5.8 years follow-up, despite a 42.5% recurrence rate. Their work particularly emphasized the value of standardized treatment protocols and rigorous follow-up in optimizing outcomes for these challenging cases.

Subgroup analyses revealed etiology-specific variations in treatment response in our meta-analysis, with success rates of 87% in myelomeningocele, 51% in spina bifida, and 84% in other neuromuscular conditions. These findings support the implementation of condition-specific prognostic counseling and treatment protocols, while affirming the feasibility and effectiveness of the Ponseti method in spina bifida populations, despite acknowledged relapse risks [37].

The observed complication rate of 28% warrants careful consideration, though most incidents comprised minor cutaneous complications manageable with meticulous casting technique—a particularly crucial consideration in patients with sensory impairments who may not recognize early signs of skin breakdown [19, 20, 28, 31, 33].

### Clinical implications

Our sensitivity analyses demonstrate that despite statistical heterogeneity, the pooled estimates provide clinically meaningful and robust guidance for therapeutic decision-making. The identification of underlying neurological condition as a key determinant of treatment success supports the development of condition-specific treatment algorithms and prognostic models. The Ponseti method emerges as a cost-effective, minimally invasive approach for managing complex foot deformities in children with neurological disorders, with optimal outcomes dependent upon early intervention, robust caregiver education and compliance, and comprehensive long-term orthotic follow-up.

These findings have particular relevance for resource-limited healthcare settings where access to complex surgical interventions may be restricted. The Ponseti method's reliance on casting expertise rather than advanced surgical facilities makes it an accessible treatment option globally, though success remains contingent upon adequate training and follow-up infrastructure.

### Limitations

This study has several methodological limitations. Substantial heterogeneity stemmed from variations in study design, patient populations, follow-up duration, and outcome definitions. Diverse neurological conditions introduced variability in treatment outcomes. Most studies were non-randomized, retrospective, with moderate sample sizes and variable quality per Newcastle–Ottawa Scale assessments [30], risking selection bias, reporting bias, and confounding. Functional outcomes were inconsistently reported, often lacking standardized tools or long-term follow-up. Heterogeneity in diagnostic criteria and treatment protocols limits generalizability. The Egger test suggested small-study effects for initial correction rates, indicating possible publication bias, though this may reflect variability in patient selection or protocol adherence. Minimal heterogeneity (I^2^ = 0.0%) supports consistent findings, with a clinically significant 90% correction rate. No publication bias was noted for recurrence rates, suggesting reliable long-term outcome reporting.

### Future directions

This systematic review and meta-analysis is the most comprehensive evaluation of the Ponseti method for neurogenic clubfoot to date. Future research should prioritize prospective, multicenter RCTs stratified by neurological diagnoses, using standardized success criteria and comprehensive outcomes over extended follow-up. We recommend structured follow-up protocols with assessments at: short-term (3, 6, 12 months), intermediate (annually until age 5), and long-term intervals (every 2–3 years until skeletal maturity), given the 52% recurrence rate. Standardized outcome measures should include: (1) objective deformity scores (Pirani/Diméglio); (2) functional assessment (Clubfoot Assessment Protocol, gait analysis when feasible) [24]; (3) radiographic evaluation (talocalcaneal angles on weight-bearing films) [11]; (4) patient-reported outcomes addressing satisfaction and brace compliance [12]; and (5) systematic complication monitoring, particularly skin issues in sensory-impaired patients [19, 28, 31]. Multicenter studies should establish centralized assessor training and standardized protocols to ensure data comparability. Investigating adjunctive therapies (e.g., botulinum toxin, dynamic bracing), developing predictive models for relapse risk, and conducting comparative effectiveness studies on bracing compliance strategies would further optimize evidence-based treatment protocols.

## Conclusion

The Ponseti method demonstrates significant efficacy in managing neurogenic clubfoot, achieving initial correction in approximately 90% of cases. However, the elevated recurrence rate of 52% necessitates structured long-term surveillance and proactive interventions to maintain correction. Given the inherent sensory impairments in this population, vigilant monitoring for cutaneous complications is essential. These findings support the Ponseti method as the preferred initial treatment approach, emphasizing the importance of individualized, multidisciplinary care and extended follow-up protocols.

## Supplementary Information

Below is the link to the electronic supplementary material.

Supplementary Material 1

Supplementary Material 2

Supplementary Material 3

Supplementary Material 4

## Data Availability

All data generated or analysed during this study are included in this published article [and its supplementary information files].

## Abbreviations

B: Bilateral
CI: Confidence interval
CTEV: Congenital talipes equinovarus
I^2^: I-squared (measure of heterogeneity)
KSA: Kingdom of Saudi Arabia
L: Left
MeSH: Medical subject headings
n: Number
NOS: Newcastle–Ottawa Scale
NR: Not reported
*p*: *p*-Value
PRISMA: Preferred reporting items for systematic reviews and meta-analyses
R: Right
τ^2^: Tau-squared
U: Unilateral
