# Supplementary material for: Effectiveness of the ponseti method in treating neurogenic clubfoot: a systematic review and meta-analysis
Source: J Orthop Surg Res. 2025 Nov 22;21:9. doi: 10.1186/s13018-025-06492-7 (PMC12777138; doi:10.1186/s13018-025-06492-7)
Supplement: Supplementary file 1 — Supplementary Material 1 [file 13018_2025_6492_MOESM1_ESM.docx]

**Supplementary Table S1:** Detailed Search Strategies for Systematic Review of the Ponseti Method in Neurological Clubfoot

**Search Date:** May 28, 2025
**Date Range:** Database inception to May 28, 2025
**Filters:** No restrictions on language, publication date, or study type were applied.

**1. PubMed (MEDLINE)**
**Platform:** PubMed
**Results:** 46 records
**Search Strategy:**

#1 "Clubfoot"[Mesh] OR "clubfoot"[tiab] OR "talipes equinovarus"[tiab] OR "club foot"[tiab]

#2 "Neuromuscular Diseases"[Mesh] OR "Cerebral Palsy"[Mesh] OR "Spinal Dysraphism"[Mesh] OR "Meningomyelocele"[Mesh] OR neurological[tiab] OR neuromuscular[tiab] OR "cerebral palsy"[tiab] OR "spina bifida"[tiab] OR "tethered cord syndrome"[tiab] OR myelomeningocele[tiab]

#3 "Ponseti method"[tiab] OR "Ponseti treatment"[tiab] OR "Ponseti"[tiab]

#4 #1 AND #2 AND #3

**2. Embase**
**Platform:** Ovid
**Results:** 65 records
**Search Strategy:**

1. exp clubfoot/ OR (clubfoot OR "talipes equinovarus" OR "club foot").ti,ab.

2. exp neuromuscular disease/ OR exp cerebral palsy/ OR exp myelomeningocele/ OR (neurological OR neuromuscular OR "cerebral palsy" OR "spina bifida" OR "tethered cord syndrome" OR myelomeningocele).ti,ab.

3. exp ponseti method/ OR ("Ponseti method" OR "Ponseti treatment" OR Ponseti).ti,ab.

4. 1 AND 2 AND 3

**3. Web of Science**

**Platform:** Clarivate

**Results:** 68 records
**Search Strategy:**

TS=("clubfoot" OR "talipes equinovarus" OR "club foot")

AND

TS=("neurological" OR "neuromuscular" OR "cerebral palsy" OR "spina bifida" OR "tethered cord syndrome" OR "myelomeningocele")

AND

TS=("Ponseti method" OR "Ponseti treatment" OR "Ponseti")

**4. Cochrane Library**
**Platform:** Wiley

**Results:** 1 record
**Search Strategy:**

("clubfoot" OR "talipes equinovarus" OR "club foot")

AND

("neurological" OR "neuromuscular" OR "cerebral palsy" OR "spina bifida" OR "tethered cord syndrome" OR "myelomeningocele")

AND

("Ponseti method" OR "Ponseti treatment" OR "Ponseti")

**5. Scopus**
**Platform:** Elsevier
**Results:** 58 records
**Search Strategy:**

TITLE-ABS-KEY("clubfoot" OR "talipes equinovarus" OR "club foot")

AND

TITLE-ABS-KEY("neurological" OR "neuromuscular" OR "cerebral palsy" OR "spina bifida" OR "tethered cord syndrome" OR "myelomeningocele")

AND

TITLE-ABS-KEY("Ponseti method" OR "Ponseti treatment" OR "Ponseti")

**Summary of Search Results**

| Database | Platform | Number of Records |
| --- | --- | --- |
| **PubMed** | PubMed | 46 |
| **Embase** | Ovid | 65 |
| **Web of Science** | Clarivate | 68 |
| **Cochrane Library** | Wiley | 1 |
| **Scopus** | Elsevier | 58 |
| **Total Records from Databases** |  | **238** |

**Additional Records Identified Through Other Sources:**
Manual screening of reference lists of included studies and relevant systematic reviews did not yield any additional eligible studies.
**Total Records for Screening:** 238

**Search Methodology Notes**

- Search strategies combined controlled vocabulary (MeSH for PubMed, Emtree for Embase) and free-text terms to maximize sensitivity.
- Boolean operators were employed with AND connecting major conceptual groups and OR within groups.
- TS = Topic Search in Web of Science (includes title, abstract, author keywords, and Keywords Plus®).
- TITLE-ABS-KEY = Title, Abstract, and Keywords field search in Scopus.
- No methodological filters or language restrictions were applied to ensure comprehensive retrieval.
- Search strategies were adapted to the specific syntax and indexing systems of each database.
