# Supplementary figures and images for: Effectiveness of the ponseti method in treating neurogenic clubfoot: a systematic review and meta-analysis
Source: J Orthop Surg Res. 2025 Nov 22;21:9. doi: 10.1186/s13018-025-06492-7 (PMC12777138; doi:10.1186/s13018-025-06492-7)

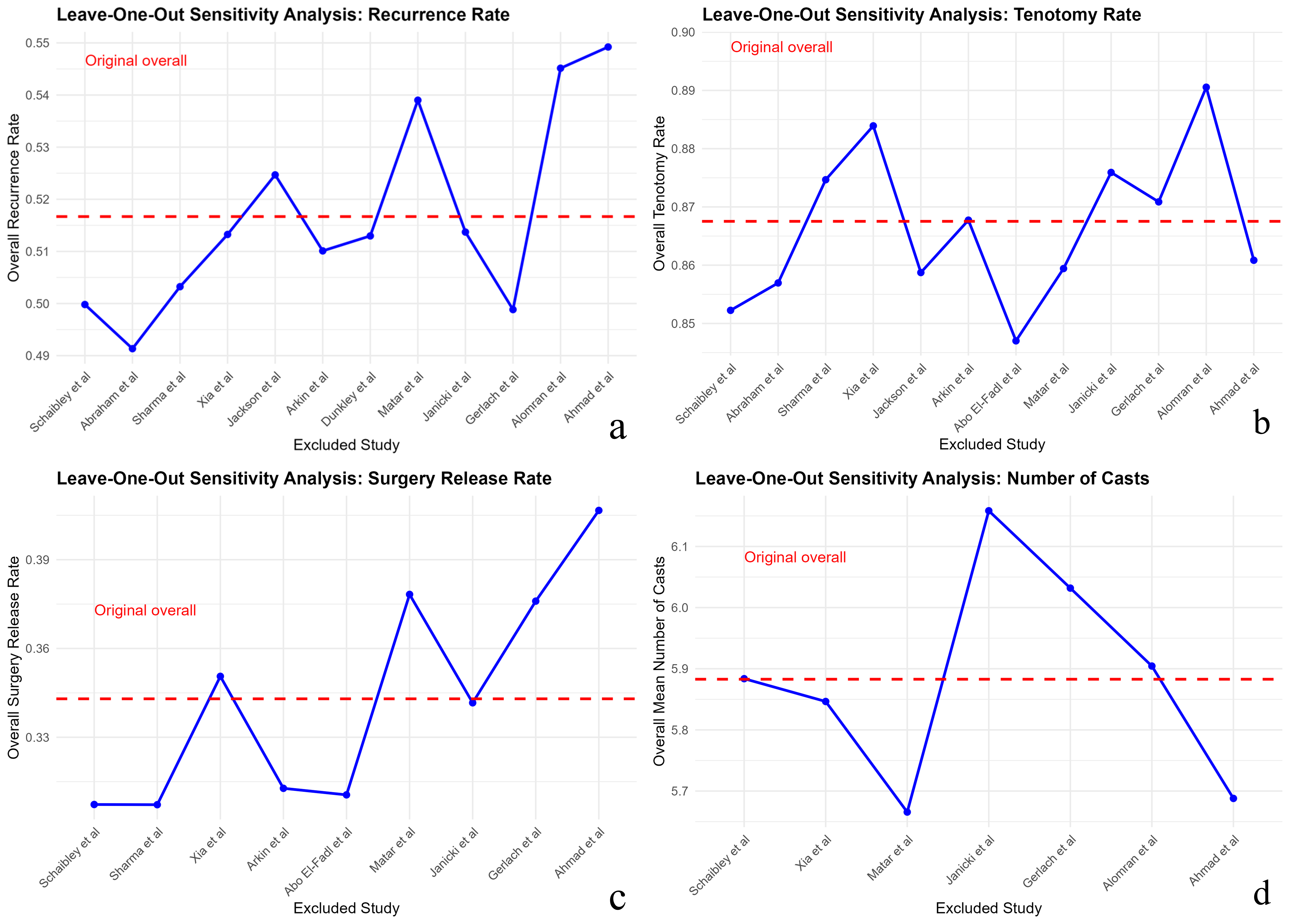

Supplement: Supplementary file 3 — Supplementary Material 3 [file 13018_2025_6492_MOESM3_ESM.tif]

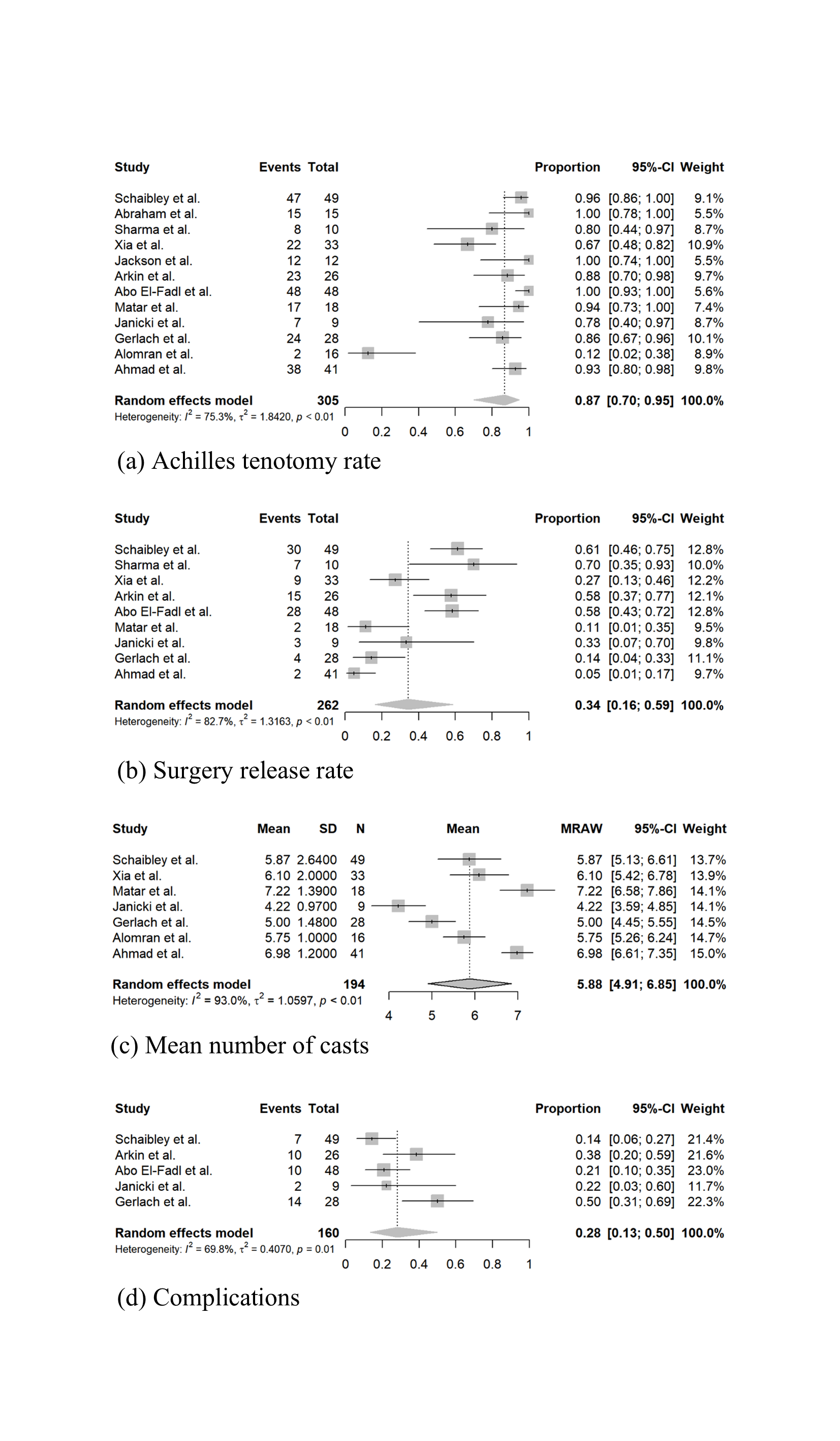

Supplement: Supplementary file 4 — Supplementary Material 4 [file 13018_2025_6492_MOESM4_ESM.tif]
